# Exploring the Two Coupled Conformational Changes That Activate the Munc18-1/Syntaxin-1 Complex

**DOI:** 10.3389/fnmol.2021.785696

**Published:** 2021-12-22

**Authors:** Jihong Gong, Xianping Wang, Chaoyang Cui, Yuyang Qin, Ziqi Jin, Cong Ma, Xiaofei Yang

**Affiliations:** ^1^Key Laboratory of Cognitive Science, Laboratory of Membrane Ion Channels and Medicine, College of Biomedical Engineering, South-Central University for Nationalities, Wuhan, China; ^2^Hubei Key Laboratory of Edible Wild Plants Conservation and Utilization, College of Life Sciences, Hubei Normal University, Huangshi, China; ^3^Key Laboratory of Molecular Biophysics of the Ministry of Education, College of Life Science and Technology, Huazhong University of Science and Technology, Wuhan, China

**Keywords:** Munc18-1, Munc13-1, syntaxin-1, synapse, exocytosis, SNARE complex

## Abstract

Calcium-dependent synaptic vesicle exocytosis is mediated by SNARE complex formation. The transition from the Munc18-1/syntaxin-1 complex to the SNARE complex is catalyzed by the Munc13-1 MUN domain and involves at least two conformational changes: opening of the syntaxin-1 linker region and extension of Munc18-1 domain 3a. However, the relationship and the action order of the two conformational changes remain not fully understood. Here, our data show that an open conformation in the syntaxin-1 linker region can bypass the requirement of the MUN NF sequence. In addition, an extended state of Munc18-1 domain 3a can compensate the role of the syntaxin-1 RI sequence. Altogether, the current data strongly support our previous notion that opening of the syntaxin-1 linker region by Munc13-1 is a key step to initiate SNARE complex assembly, and consequently, Munc18-1 domain 3a can extend its conformation to serve as a template for association of synaptobrevin-2 and syntaxin-1.

## Introduction

Neurotransmitter release is mediated by the calcium-dependent exocytosis of synaptic vesicles (Südhof and Rizo, 2011). Synaptic vesicles loaded with neurotransmitters are transported to the presynaptic active zone, docked at the presynaptic membrane, and primed for release in an ATP-dependent manner. Upon Ca^2+^ influx, the vesicles fuse with the presynaptic membrane to release neurotransmitters into the synaptic cleft (Südhof and Rizo, 2011; Jahn and Fasshauer, 2012). The core fusion machinery for synaptic exocytosis comprises three SNAREs (soluble N-ethylmaleimide-sensitive-factor-attached protein receptors), including syntaxin-1, synaptobrevin-2, and SNAP-25 (Brunger, 2005; Jahn and Scheller, 2006). The SNAREs form a tight SNARE complex, consisting of a four-helix bundle, to bring the two membranes together and drive membrane fusion (Sutton et al., 1998; Weber et al., 1998; Stein et al., 2009). To achieve the exact regulation of exocytosis, numbers of other SNARE regulatory proteins are required, including Munc18-1 and Munc13-1 (Malsam et al., 2008; Jahn and Fasshauer, 2012; Rizo and Xu, 2015).

In neurons, deficiency of Munc18-1 or Munc13s impedes the release of neurotransmitters (Augustin et al., 1999; Richmond et al., 1999; Verhage et al., 2000). Munc18-1 binds tightly to syntaxin-1 to form the Munc18-1/syntaxin-1 complex (Misura et al., 2000; Burkhardt et al., 2008; Colbert et al., 2013), which chaperones syntaxin-1 to the plasma membrane (Arunachalam et al., 2008; Han et al., 2009). However, in this complex, Munc18-1 locks syntaxin-1 in a “closed” conformation, preventing syntaxin-1 from entering the SNARE complex (Dulubova et al., 1999; Yang et al., 2000). This might lead to the accumulation of vesicle in the docking stage (Toonen et al., 2006; Han et al., 2011). In the vesicle priming process, Munc13-1 catalyzes the transition from the Munc18-1/syntaxin-1 complex to the SNARE complex in the presence of synaptobrevin-2 and SNAP-25 (Ma et al., 2011). The NF (N1128, F1131) sequence in the middle of the Munc13-1 MUN domain interacts with the RI (R151, I155) sequence in the syntaxin-1 linker region (Yang et al., 2015; Wang et al., 2017). During the transition, two conformational changes occur, the opening of the syntaxin-1 linker region and the extension of Munc18-1 domain 3a (Misura et al., 2000; Margittai et al., 2003; Parisotto et al., 2014; Munch et al., 2016; Wang et al., 2017). Previous studies have shown that either a L165A/E166A (LEAA) mutation of syntaxin-1 that helps to open its linker region or a P335A mutation of Munc18-1 that promotes its domain 3a extension can facilitate vesicle exocytosis in neurons and neuroendocrine cells (Dulubova et al., 1999; Gerber et al., 2008; Martin et al., 2013; Han et al., 2014; Munch et al., 2016; Park et al., 2017). Interestingly, in *C. elegans*, overexpression of the syntaxin-1 LEAA or Munc18-1 P335A variants rescued vesicle exocytosis defects caused by deletion of *unc13*, the invertebrate homolog of Munc13s (Richmond et al., 2001; Hammarlund et al., 2007; Park et al., 2017), suggesting that the conformational changes of the syntaxin-1 linker region and Munc18-1 domain 3a are highly related with the function of Munc13-1. At least two questions are raised: which conformational change represents the decisive step in the transition of the Munc18-1/syntaxin-1 complex to the SNARE complex, and how do the two conformational changes couple? In previous *in vitro* studies, we have found that both the syntaxin-1 LEAA mutant and the Munc18-1 P335A mutant could initiate the transition of the Munc18-1/syntaxin-1 complex to the SNARE complex in the absence of the MUN domain (Yang et al., 2015; Wang et al., 2020). In addition, our *in vitro* results suggest that the opening of syntaxin-1 linker region driven by the MUN domain leads to the extension of domain 3a (Wang et al., 2020). However, the physiological relevance of our conclusions derived from *in vitro* data has not been tested.

In this study, we investigate the relationship between the two conformational changes using *in vivo* electrophysiological experiments and *in vitro* FRET and native-PAGE assay. Our results confirmed the notion that Munc13-1 activates the Munc18-1/syntaxin-1 complex via driving the two conformational changes, wherein opening of the syntaxin-1 linker region by the Munc13-1 MUN domain result in the extension of Munc18-1 domain 3a. Taken together, our data strongly suggest that opening of the syntaxin-1 linker region by Munc13-1 is a key step to initiate SNARE complex assembly.

## Materials and Methods

### Plasmid Construction

The shRNAs of syntaxin-1, Munc13-1, and Munc18-1 were used as previously reported (Zhou et al., 2013; Wang et al., 2017, 2020). To knockdown the syntaxin-1a and 1b, the shRNA oligonucleotides (sense sequences: TCGACCAGAGGCAGCTGGAGATCACTTCAAGAGAGTGA TCTCCAGCTGCCTCTGGTTTTTTGGAAAT and CGCGCC CGATCATCATTTGCTGTGTGTTCAAGAGACACACAGCAA ATGATGATCATTTTTTTGGAAA) were designed to target two conserved regions, and inserted into *Xho*I/*Xba*I and *Asc*I/*Rsr*II sites of lentiviral vector L309, respectively. The shRNA oligonucleotides (sense sequences: GTCTGT CCACTCTCTCATCCCATGGGATGAGAGAGTGGACAGAC) were inserted into *Xho*I/*Xba*I sites of L309 to silence the Munc18-1 expression. To silence the expression of Munc13-1, the shRNA oligonucleotides (sense sequences: CCGGCCCGTGTGAAACAAAGGTTTACTCGAGTAAACCT TTGTTTCACACGGGTTTTT) were inserted into *Xho*I/*Xba*I sites of L309. Munc18-1 and its mutant sequences, syntaxin-1 and its mutant sequences, or Munc13-1 C1C2BMUN domain and its mutant sequences were inserted into the *Bam*HI/*Eco*RI sites.

Rat Munc18-1 (residues 1–594), Munc18-1 mutants P335A, KKEE (K332E K333E), and rat syntaxin-1, syntaxin-1 mutants LEAA, RIAA were cloned into pGEX-KG vector that includes an N-terminal GST tag. Full-length rat synaptobrevin-2 were cloned into pGEX-6p-1 vector. Rat Mun13-1 MUN domain were constructed into pET28a vector.

### Cell Culture

HEK293T cells (CRL-11268, ATCC) were cultured in Dulbecco’s modified Eagle’s medium (Gibco), supplemented with 10% (v/v) fetal bovine serum and penicillin-streptomycin (50 and 50 μg/mL).

Neuron cultures were obtained from the cortex of newborn Kunming mice as described previously (Wang et al., 2019). Briefly, the cerebral cortex was dissected from postnatal day 0 (P0) mice, digested with 0.25% trypsin at 37^°^C for 12 min, blown and sucked into single cells. The single cell suspension was obtained after passing the cell strainer. Neurons were planted on glass coverslips coated with poly-L-lysine and cultured in the medium MEM (Gibco) supplemented with 0.5% (w/v) glucose, 100 mg/L transferrin, 5% (v/v) fetal bovine serum, 2% (v/v) B27 (Gibco), and 2 mM Ara-C (Sigma). The culture medium was changed on DIV (day *in vitro*) 1, 4 and 9, in the manner of 500 μL out and 600 μL in. Neurons were infected with lentiviruses at DIV 5–7, and analyzed at DIV13–14.

Neurons and HEK293T cells were all grown at 37^°^C, 5% CO_2_ in a cell incubator (Thermo).

### Lentivirus Preparation

Lentivirus preparation from HEK293T cells as described previously. In brief, lentivirus vectors (L309 or its constructions) and three helper plasmids (pRSV-REV, pMDLg-pRRE, and pVSVG) were co-transfected by polyethylenimine (PEI) into HEK293T cells. The ratio of each component was L309: pRSV-REV: pMDLg-pRRE: pVSVG: PEI = 3: 2: 2: 1: 24. The plasmids and PEI were in the medium Opti-MEM (Gibco), incubated for 30 min at room temperature, and added into the HEK293T cells. The cell culture medium containing virus was harvested at 48 h after transfection, and then precleaned with 1,000 *g* centrifuge. The virus-containing medium was overlaid on a sucrose-containing buffer (50 mM Tris–HCl pH 7.4, 100 mM NaCl, 0.5 mM EDTA) at 4:1 (v/v), and centrifuged in 10,000 *g* for 4 h at 4^°^C (Jiang et al., 2015). After centrifugation, the buffer and medium were removed. PBS buffer was added into the tube carefully and incubated in a 4^°^C fridge overnight for virus resuspension. All steps were carried out under level II biosafety conditions.

### Electrophysiological Recording

Electrophysiological recording was performed in whole-cell patch clamping mode by using HEKA EPC10 amplifier as described previously (Gong et al., 2016). Micropipettes were pulled from borosilicate glass capillary tubes (Word Precision Instruments) by using P-97 puller (Sutter instruments). The micropipette solution contained 120 mM CsCl, 10 mM HEPES, 10 mM EGTA, 0.3 mM Na-GTP, 3 mM Mg-ATP. Adjust the pH to 7.2–7.4 with CsOH, and adjust the osmotic pressure with dd H_2_O to about 305. Add 5 mM QX-314 to the micropipette solution before use for the evoke release recording. The bath solution contained 140 mM NaCl, 5 mM KCl, 2 mM MgCl_2_, 2 mM CaCl_2_, 10 mM HEPES, and 10 mM glucose. Adjust the pH to 7.2–7.4 with NaOH, and adjust the osmotic pressure with dd H_2_O to about 315. Inhibitory postsynaptic currents (IPSCs) were isolated by adding the AMPA and NMDA receptor blockers CNQX (20 μM) and AP-5 (50 μM) to the bath solution. Evoked IPSC was recorded in the stimulus pulse (90 μA) mini IPSCs were recorded in the presence of tetrodotoxin (1 μM) for action-potential blocking. Sucrose IPSC was measured with a 0.5 M sucrose application in the bath solution. Electrophysiological data analyzed by Clampfit 10 (Molecular devices).

### Protein Expression and Purification

All recombinant proteins were expressed in *Escherichia coli* BL21 (DE3) and purified as described previously (Wang et al., 2020). In short, the cells were grown to the OD_600_ ≈ 0.8 at 37^°^C and induced by 0.5 mM IPTG (isopropyl-β-thiogalactoside) at 16 or 20^°^C. Cultured cells were harvested by centrifugation at 5,000 *g*, 4^°^C for 20 min, resuspended by PBS, and lysed with high pressure crusher in the presence of 1 mM PMSF (phenylmethanesulfonyl fluoride), 5 mM DTT (1,4-dithio-DL-threitol), and 1 mM EDTA (pH 8.0), then centrifugated at 31,000 *g*. For the GST-fused proteins, the supernatants were collected and mixed with glutathione Sepharose 4B (GE Healthcare) affinity media. Thrombin (Sigma) was used for removing GST fusion tag. For hexa-histidine-tagged proteins, the supernatants were collected and mixed with Nickel-NTA agarose (Qiagen) affinity media, and eluted with buffer containing 300 mM imidazole. The eluted proteins were further purified by size-exclusion chromatography. All recombinant proteins were expressed in Escherichia coli BL21 (DE3) and purified as described previously (Wang et al., 2020). In short, the cells were grown to the OD600 ≈ 0.8 at 37^°^C and induced by 0.5 mM IPTG (isopropyl-β-thiogalactoside) at 16 or 20^°^C. Cultured cells were harvested by centrifugation at 5,000 *g*, 4^°^C for 20 min. For GST-fused proteins, cells were resuspended by PBS (pH 7.4) supplemented with 1 mM PMSF (phenylmethanesulfonyl fluoride), 5 mM DTT (1,4-dithio-DL-threitol), and 1 mM EDTA (pH 8.0), lysed with high pressure crusher and then centrifugated at 31,000 *g*. the supernatants were collected and mixed with glutathione Sepharose 4B (GE Healthcare) affinity media. Thrombin (Sigma) was used for removing GST tag. For hexa-histidine-tagged proteins, cells were resuspended with Tris–HCl buffer (50 mM Tris–HCl pH 8.0, 150 mM NaCl, 10% glycerol) containing 5 mM imidazol. Cells were lysed and centrifugated as GST-fused proteins. The supernatants were incubated with Nickel-NTA agarose (Qiagen) affinity media. Hexa-histidine-fused proteins bound to agarose affinity media were eluted with Tris–HCl buffer buffer containing 300 mM imidazole. Both eluted proteins were further purified by size-exclusion chromatography.

### Native PAGE

Native gel was prepared with polyacrylamide without SDS. To obtain the Munc18-1/syntaxin-1 complex, purified Munc18-1 and syntaxin-1 were incubated together in the ratio of 1.2:1 at 4^°^C overnight. Then, the Munc18-1/syntaxin-1 (3 μM/2.5 μM) complex was mixed with synaptobrevin-2 (10 μM) and SNAP-25 (10 μM), with or without Munc13-1 MUN domain (30 μM) at 30^°^C for 2 h. Then samples were mixed with 5 × loading buffer and electrophoresis was carried out at 4^°^C for 2 h in 15% native gel. Gels were stained with Coomassie Brilliant Blue.

### Ensemble FRET Assay

Purified synaptobrevin-2 (residues 29–93, with mutation of S61C) and SNAP-25 (with native cysteines mutated to serine and mutation of Q197C) were mixed and labeled with donor-dye BODIPY FL-maleimide (Molecular Probes) and acceptor dye tetramethylrhodamine-5-maleimide. The ratio of protein and dye was 1:5. The mixture rotated overnight at 4^°^C avoiding light in buffer containing 25 mM HEPES (pH 7.4), 150 mM KCl, and 10% (v/v) glycerol. Excess dyes were removed by PD-10 desalting columns (GE Healthcare) after addition of 10 mM DTT. To obtain the Munc18-1/syntaxin-1 or its mutant complex, Munc18-1, or its mutants P335A/KKEE and syntaxin-1 or its mutants LEAA/RIAA were pre-incubated with a ratio of 1.5:1 at 4^°^C overnight. The labeled synaptobrevin-2 (2 μM) and SNAP-25 (10 μM) were mixed with Munc18-1/syntaxin-1 (5 μM) or its mutant complex (5 μM) and Munc13-1 MUN domain (30 μM). All ensemble FRET experiments were performed by a QM 40 spectrofluorometer (Photon Technology Incorporated) in a 1-cm quartz cuvette at 30^°^C. The excitation and emission wavelength were 485 and 513 nm. FRET efficiency was calculated by the formula: *E* = (*F*_0_-*F*_obs_)/*F*_0_ × 100% (*E*: FRET efficiency; *F*_0_: the initial fluorescent intensity; *F*_obs_, the observed fluorescent intensity).

### Statistical Analysis

Prism 6.01 (GraphPad) was used for statistical analysis, all of which are described in figure legends.

## Results

### Syntaxin-1 LEAA Bypasses the Requirement for Munc13-1 NF Sequence in Vesicle Exocytosis

**FIGURE 1:**
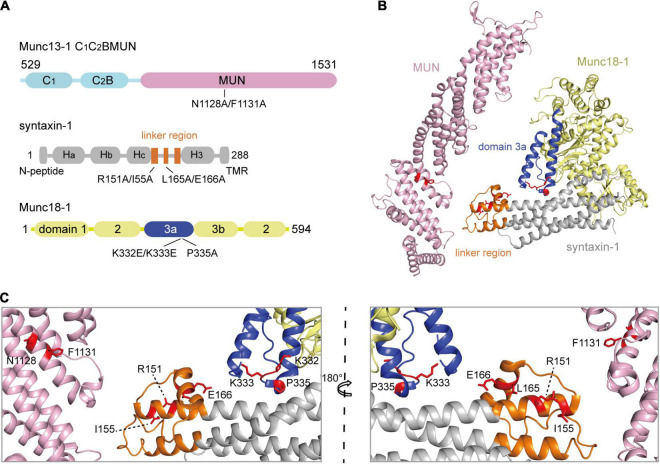
The schematic diagrams show the crucial sequences of Munc13-1, Munc18-1, and syntaxin-1. **(A)** Domain depictions of Munc13-1 C1C2BMUN, syntaxin-1, and Munc18-1. Mutations used in this paper are shown below, including N1128A/F1131A on C1C2BMUN domain, R151A/I155A or L165A/E166A on the syntaxin-1 linker region, and K332E/K333E or P335A on the Munc18-1 domain 3a. TMR, transmembrane region. **(B)** Crystal structure overview of MUN domain (PDB ID 4Y21) and Munc18-1/syntaxin-1 (PDB ID 3C98). Munc18-1 domain 3a and syntaxin-1 linker region are shown in blue and orange, respectively. Mutating residues are highlighted with red sticks. **(C)** Enlarged views of labels correspond to residues that were mutated. Two orientations rotating 180° with respect to each other are shown.

In our previous *in vitro* studies, we showed that the opening of synatxin-1 linker region and the extension of Munc18-1 domain 3a are driven by the interaction of the Munc13-1 MUN domain with the Munc18-1/syntaxin-1 complex (Wang et al., 2017, 2020). To verify this *in vivo*, we employed the crucial mutations that were previously identified (Figure 1), including N1128A/F1131A in Munc13-1 MUN domain (Yang et al., 2015), R151A/I155A and L165A/E166A in the syntaxin-1 linker region (Dulubova et al., 1999; Wang et al., 2017), and K332E/K333E and P335A in the Munc18-1 domain 3a (Han et al., 2014; Parisotto et al., 2014; Wang et al., 2020). The NF sequence in the MUN domain and RI sequence in the syntaxin-1 linker region correspond to the essential binding sites responsible for the MUN domain targeting to the syntaxin-1 linker region (Figure 1C). The syntaxin-1 LEAA mutation helps to open the syntaxin-1 linker region (Wang et al., 2017) and the Munc18-1 P335A mutation promotes the extension of domain 3a (Parisotto et al., 2014).

**FIGURE 2:**
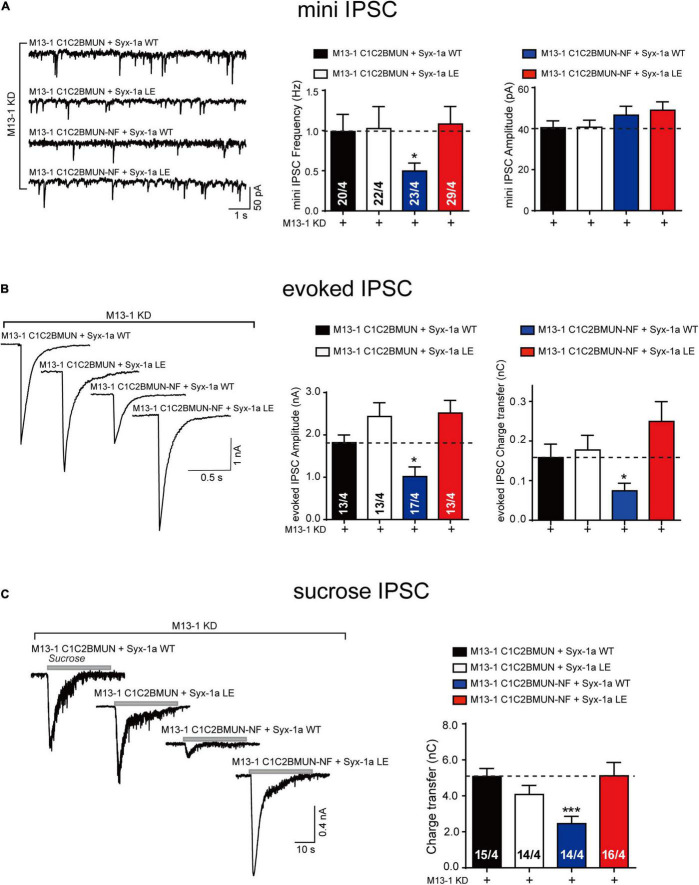
The syntaxin-1 LEAA mutant bypasses the requirement of Munc13-1 NFAA mutation in synaptic vesicle exocytosis. **(A)** Sample traces (left), summary graphs of frequency (middle) and amplitude (right) of mini IPSC recorded from cultured cortical neurons that were infected with lentivirus expressing the Munc13-1 shRNAs (M13-1 KD), Munc13-1 C1C2BMUN or its NFAA mutant (M13-1 C1C2BMUN-NF), and wild-type syntaxin-1a (Syx-1a WT) or its LEAA mutant (Syx-1a LE). **(B)** Sample traces (left), summary graphs of amplitude (middle), and charge transfer (right) of evoked IPSC recorded from neurons that are described in **(A)**. **(C)** Sample traces (left), summary graphs of charge transfer (right) of sucrose evoked IPSC recorded from neurons that described in **(A)**. Data information: Numbers of cells/independent cultures analyzed are listed in the bars. Data shown in summary graphs are mean values ± SEM. Statistical significance was analyzed by Student’s *t*-test, *p < 0.05; ***p < 0.001.

We used a knockdown (KD)-rescue approach in cultured mouse cortical neurons. The endogenous expression of Munc13-1 and syntaxin-1 (syntaxin-1a and 1b) was suppressed by lentivirus-delivered short hairpin RNAs (shRNAs), as previously reported (Zhou et al., 2013; Wang et al., 2017). We then monitored synaptic exocytosis with the expression of the main functional domain of Munc13-1, C1C2BMUN, or its NFAA mutant, as well as syntaxin-1a or its LEAA mutant. Expression of C1C2BMUN and syntaxin-1a rescued both the frequency of spontaneous mini IPSCs and the amplitude and charge transfer of action potential-evoked IPSCs (Figures 2A,B). In contrast, the NFAA mutant of C1C2BMUN cannot rescue any of these parameters (Figures 2A,B), implying that the NFAA mutant fails to support synaptic vesicle exocytosis, consistent with previous results (Wang et al., 2017). Interestingly, the expression of the syntaxin-1 LEAA mutant rescued the defects caused by the C1C2BMUN NFAA mutation, suggesting that the opening of the syntaxin-1 linker region bypasses the role of the Munc13-1 NF sequence in both spontaneous and evoked release. In addition, the mini IPSC amplitude was unchanged under all conditions (Figure 2A), ruling out major postsynaptic effects of these mutant proteins.

We next analyzed vesicle priming by characterizing the size of the readily releasable pool of vesicles induced by the application of a hypertonic sucrose solution (Rosenmund and Stevens, 1996). The significant decrease in the sucrose-induced charge transfer observed in neurons expressing the C1C2BMUN NFAA mutant was rescued by expressing the syntaxin-1 LEAA mutant but not by expressing wild-type (WT) syntaxin-1 (Figure 2C). Therefore, these *in vivo* data indicate that the open form of syntaxin-1 linker region (syntaxin-1 LEAA) can compensate the function of the Munc13-1 NF sequence in priming of synaptic vesicles.

### Syntaxin-1 LEAA Is Equal to the Function of the MUN NF Sequence in SNARE Assembly

**FIGURE 3:**
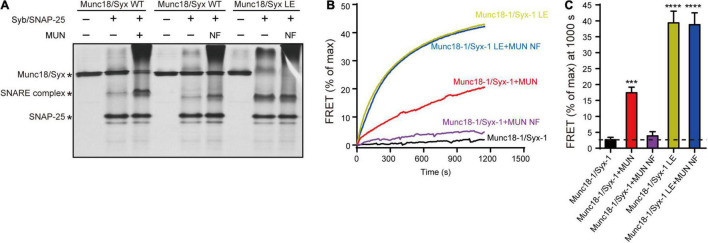
The syntaxin-1 LEAA mutant is equal to the function of MUN NFAA mutation in SNARE assembly. **(A)** The SNARE complex assembly from Munc18-1/syntaxin-1 (Munc18/Syx WT) or Munc18-1/syntaxin-1 LEAA mutant (Munc18/Syx LE) with addition of synaptobrevin-2 (Syb), SNAP-25, MUN domain or MUN NFAA mutant (NF) detected by native gel. **(B)** The SNARE complex assembly from Munc18-1/syntaxin-1 (Munc18-1/Syx-1 WT) or Munc18-1/syntaxin-1 LEAA mutant (Munc18-1/Syx-1 LE), in the absence or presence of synaptobrevin-2, SNAP-25, MUN domain or MUN NFAA mutant (MUN NF) detected by FRET assay. FRET between the BODIPY FL-labeled synaptobrevin-2 (donor) and TMR-labeled SNAP-25 (acceptor) was monitored. **(C)** Summary graphs of FRET efficiency calculated from **(A)**. Data shown in summary graphs are mean values ± SD, *n* = 3. Statistical significance was analyzed by Student’s *t*-test, ***p < 0.001; ****p < 0.0001.

We thought to verify the above results by using a previously established native-PAGE assay that can detect the transition from the Munc18-1/syntaxin-1 complex to the SNARE complex catalyzed by the MUN domain (Wang et al., 2020). Consistent with previous results, the transition to the SNARE complex assembly from the Munc18-1/syntaxin-1 complex was promoted by the MUN domain but not by the MUN NFAA mutant in the presence of synaptobrevin-2 and SNAP-25 (Figure 3A). However, when the Munc18-1/syntaxin-1 LEAA mutant was used, the transition can be promoted the MUN NFAA mutant (Figure 3A), suggesting that the open form of syntaxin-1 compensate the function of the NF sequence in SNARE assembly.

We further characterized the function of the syntaxin-1 LE sequence using an ensemble FRET assay (Wang et al., 2017). The FRET experiments demonstrated that the NFAA mutation abrogated the MUN-catalyzed transition to the SNARE complex from the Munc18-1/syntaxin-1 complex (Figures 3B,C). Likewise, the syntaxin-1 LEAA mutant promoted the transition to the SNARE complex by rescuing the defects caused by the NFAA mutation (Figures 3B,C).

Collectively, our data confirmed that the MUN domain is crucial for synaptic vesicle release due to the functional importance of its NF sequence in the transition from the Munc18-1/syntaxin-1 complex to the SNARE complex. Moreover, our results showed that the open form of the syntaxin-1 linker region was equal to the function of the Munc13-1 NF sequence, emphasizing that syntaxin-1 linker region opening is driven by the Munc13-1 MUN interaction with Munc18-1/syntaxin-1.

### An Extension State of Munc18-1 Domain 3a Overcomes the Defect of the Syntaxin-1 Linker Region Opening in Vesicle Exocytosis

**FIGURE 4:**
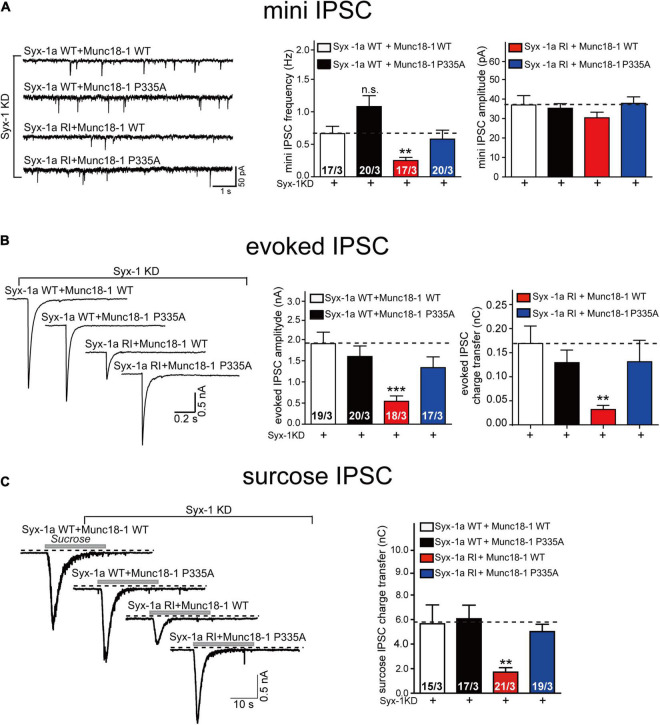
The Munc18-1 P335A mutant supports synaptic vesicle exocytosis in neurons expressing syntaxin-1 RIAA mutant. **(A)** Sample traces (left), summary graphs of frequency (middle) and amplitude (right) of mini IPSC recorded from cultured cortical neurons that were infected with lentivirus expressing the syntaxin-1 shRNAs (Syx-1 KD), wild-type syntaxin-1a (Syx-1a WT) or its RIAA mutant (Syx-1a RI), and wild-type Munc18-1 (Munc18-1WT) or its P335A mutant (Munc18-1 P335A). **(B)** Sample traces (left), summary graphs of amplitude (middle), and charge transfer (right) of evoked IPSC recorded from neurons that are described in **(A)**. **(C)** Sample traces (left), summary graphs of charge transfer (right) of sucrose evoked IPSC recorded from neurons that described in **(A)**. Data information: Numbers of cells/independent cultures analyzed are listed in the bars. Data shown in summary graphs are mean values ± SEM. Statistical significance was analyzed by Student’s *t*-test, **p < 0.01; ***p < 0.001.

Two conformational changes occur in the Munc18-1/syntaxin-1 complex upon its activation by Munc13-1. However, the relationship between the two conformational changes under physiological conditions has not been fully understood. Our previous *in vitro* studies have shown that the opening of syntaxin-1 linker region driven by the MUN domain leads to the extension of Munc18-1 domain 3a (Wang et al., 2020). The MUN domain was unable to open the linker region when syntaxin-1 contains the RIAA mutation, as the RI sequence mediates interaction with the MUN NF pocket (Wang et al., 2017). To further figure out the action order of the two conformational changes, we suppressed the endogenous expression of Munc18-1 and syntaxin-1 (syntaxin-1a and 1b) using lentivirus-delivered shRNAs in mouse cortical neurons. We found that the expression of the syntaxin-1 RIAA mutant decreased both the frequency of mini IPSCs and the amplitude and charge transfer of evoked IPSCs (Figures 4A,B), which is consistent with previous findings (Wang et al., 2017). Interestingly, this defect was rescued by the expression of Munc18-1 P335A, but not by Munc18-1 WT (Figures 4A,B). These results imply that Munc18-1 P335A bypasses the opening of syntaxin-1 linker region in synaptic vesicle exocytosis.

In addition, we analyzed vesicle priming, as accessed by measurements of the readily releasable vesicle pool (Figure 4C). As before, the decrease in sucrose-induced charge transfer in neurons expressing the RIAA mutant was rescued by expressing Munc18-1 P335A but not Munc18-1 WT (Figure 4C), indicating that Munc18-1 P335A could overcome the defect of opening of sytanxin-1 linker region in the priming of synaptic vesicles. Thus, our results suggest that the extension of domain 3a actions downstream of syntaxin-1 linker region opening. Our data confirm that the extension of Munc18-1 domain 3a results from of the interaction of the Munc18-1/syntaxin-1 complex with Munc13-1 MUN domain.

### An Extension State of Munc18-1 Domain 3a Rescues the Defect of the Syntaxin-1 Linker Region Opening in SNARE Assembly

**FIGURE 5:**
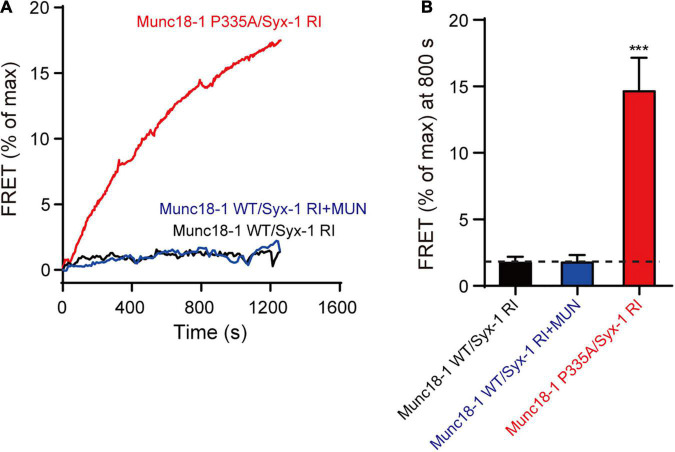
The Munc18-1 P335A rescues the defect of syntaxin-1 RIAA mutant in SNARE assembly. **(A)** The SNARE complex assembly from Munc18-1/syntaxin-1 RIAA mutant (Munc18-1 WT/Syx-1 RI) or Munc18-1 P335A mutant/syntaxin-1 RIAA mutant (Munc18-1 P335A/Syx-1 RI), synaptobrevin-2, SNAP-25, with or without MUN domain detected by FRET assay. FRET between the BODIPY FL-labeled synaptobrevin-2 (donor) and TMR-labeled SNAP-25 (acceptor) was monitored. **(B)** Summary graphs of FRET efficiency calculated from **(A)**. Data shown in summary graphs are mean values ± SD, *n* = 3. Statistical significance was analyzed by Student’s *t*-test, ***p < 0.001.

To corroborate the above results, we used native-PAGE and ensemble FRET assays to analyses the MUN-catalyzed transition from the Munc18-1/syntaxin-1 complex to the SNARE complex. Consistent to previous results, the syntaxin-1 RIAA mutant abolished SNARE complex formation in the presence of MUN (Figure 5 and Supplementary Figure 1), confirming that RI residues are crucial for the MUN-catalyzed transition to the SNARE complex. However, the SNARE complex formed when Munc18-1 P335A was complexed with the syntaxin-1 RIAA mutant (Figure 5 and Supplementary Figure 1), indicating that the defect caused by syntaxin-1 RIAA was rescued by Munc18-1 P335A. Our results suggest that the extension of domain 3a bypasses the requirement for opening of syntaxin-1 linker region in SNARE complex assembly.

In summary, the above results suggest that the extension of Munc18-1 domain 3a and the opening of the syntaxin-1 linker region are caused by the interaction between MUN and Munc18-1/syntaxin-1. The extension of domain 3a is a subsequent event following the opening of syntaxin-1 linker region.

### Syntaxin-1 LEAA Rescues the Munc18-1 Domain 3a KKEE Mutation in Vesicle Exocytosis and SNARE Assembly

Synaptic exocytosis and SNARE complex assembly can occur when Munc18-1 domain 3a is extended (P335A mutant, gain-of-function) even when the syntaxin-1 linker region adopts a closed conformation (RIAA mutant, loss-of-function). However, when the syntaxin-1 linker region is in a constitutive opened state (LEAA, gain-of-function), it is unclear whether the state of Munc18-1 domain 3a influences synaptic exocytosis. According to previous results, the KKEE mutation in domain 3a abolishes the vesicle exocytosis because of its inability to interact with the H3 domain of syntaxin-1 which impedes the Munc18-1 function in templating SNARE complex assembly. Hence, we use the Munc18-1 KKEE mutant and the syntaxin-1 LEAA mutant to address this issue.

**FIGURE 6:**
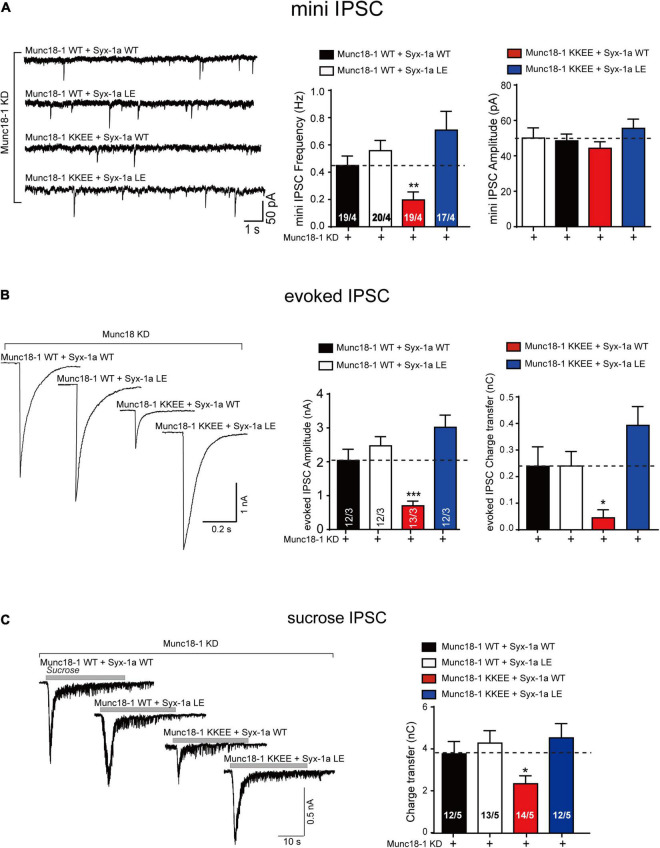
The syntaxin-1 LEAA mutant rescues the KKEE mutation of Munc18-1 in synaptic vesicle release. **(A)** Sample traces (left), summary graphs of frequency (middle) and amplitude (right) of mini IPSC recorded from cultured cortical neurons that were infected with lentivirus expressing the Munc18-1 shRNAs (Munc18-1 KD), wild-type Munc18-1 (Munc18-1 WT) or its KKEE mutant (Munc18-1 KKEE), and wild-type syntaxin-1a (Syx-1a WT) or its LEAA mutant (Syx-1a LE). **(B)** Sample traces (left), summary graphs of amplitude (middle), and charge transfer (right) of evoked IPSC recorded from neurons that described in **(A)**. **(C)** Sample traces (left), summary graphs of charge transfer (right) of sucrose evoked IPSC recorded from neurons that described in **(A)**. Data information: Numbers of cells/independent cultures analyzed are listed in the bars. Data shown in summary graphs are mean values ± SEM. Statistical significance was analyzed by Student’s *t*-test, *p < 0.05; **p < 0.01; ***p < 0.001.

**FIGURE 7:**
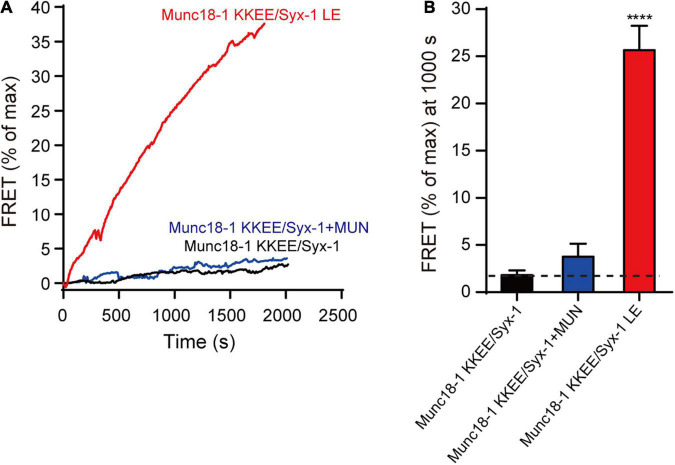
The syntaxin-1 LEAA mutant overcomes the KKEE mutation of Munc18-1 in SNARE assembly. **(A)** The SNARE complex assembly from Munc18-1 KKEE mutant/syntaxin-1 (Munc18-1 KKEE/Syx-1) or Munc18-1 KKEE mutant/syntaxin-1 LEAA mutant (Munc18-1 KKEE/Syx-1 LE), synaptobrevin-2, SNAP-25, with or without MUN domain detected by FRET assay. FRET between the BODIPY FL-labeled synaptobrevin-2 (donor) and TMR-labeled SNAP-25 (acceptor) was monitored. **(B)** Summary graphs of FRET efficiency calculated from **(A)**. Data shown in summary graphs are mean values ± SD, *n* = 3. Statistical significance was analyzed by Student’s *t*-test, ****p < 0.0001.

The endogenous expression of Munc18-1 and syntaxin-1 (syntaxin-1a and 1b) was suppressed by lentivirus-delivered shRNAs in mouse cortical neurons, as described above. The Munc18-1 KKEE mutant, which abolished the template function of domain 3a, was unable to rescue the decrease in the mini IPSC frequency, the evoked IPSC amplitude, or the evoked charge transfer. Overexpression of the syntaxin-1 LEAA mutant rescued the decrease in the mini IPSC frequency and evoked IPSC amplitude and charge transfer caused by Munc18-1 KKEE (Figures 6A,B). We also analyzed vesicle priming (Figure 6C). Likewise, the decrease in sucrose-evoked charge transfer caused by the Munc18-1 KKEE mutant was rescued by expressing the syntaxin-1 LEAA mutant, but not by syntaxin-1 WT (Figure 6C), indicating that the opening of the syntaxin-1 linker region promotes vesicle priming in a way that is not dependent on binding of domain 3a to the syntaxin-1 H3, which is not expected. To address this *in vitro*, we further conducted a SNARE complex assembly experiment using FRET assay. The KKEE mutant of Munc18-1 abolished the transition of Munc18-1/syntaxin-1 to the SNARE complex catalyzed by MUN (Figure 7). However, the syntaxin-1 LEAA mutant promoted the formation of the SNARE complex by rescuing the defects caused by the KKEE mutation (Figure 7). A plausible explanation is that the syntaxin-1 LEAA combined with the Munc18-1 KKEE mutation exerts a strong effect leading to the escape of the syntaxin-1 H3 from Munc18-1 clamping. In this circumstance, the H3 assembles with synaptobrevin-2 and SNAP-25 to form the SNARE complex in a manner independent of Munc18-1 and Munc13-1.

## Discussion

Previous studies have shown that two conformational changes in the Munc18-1/syntaxin-1 complex are involved in the transition to the SNARE complex, including (i) transition of the linker region of syntaxin-1 from a defined structure to a random coil (Misura et al., 2000; Margittai et al., 2003; Wang et al., 2017) and (ii) transition of domain 3a of Munc18-1 from a bent to an extended conformation (Parisotto et al., 2014; Munch et al., 2016; Jiao et al., 2018). The two conformational changes are mediated by the MUN domain that interacts with Munc18-1/syntaxin-1. Q301/K308 residues on the domain 3a of Munc18-1 and residues R151/I155 on the syntaxin-1 linker region collaborate to provide the interaction sites for the MUN domain (Wang et al., 2017, 2020). In this study, we investigated the relationship between these two conformational changes using both *in vitro* FRET and native-PAGE and *in vivo* electrophysiological assays.

First, we investigated the relationship between the syntaxin-1 linker region and the Munc13-1 MUN domain and found that the constitutively open form of syntaxin-1 is equal to the function of the MUN NF sequence in the process of SNARE assembly and vesicle exocytosis (Figures 2, 3). Our results further confirmed that the opening of the syntaxin-1 linker region is driven by Munc13-1 MUN interacting with the Munc18-1/syntaxin-1 complex (Figures 2, 3). A previous study reported that in *C. elegans*, syntaxin-1 LEAA could partially rescued the knockout of *unc-13* (Richmond et al., 2001). Here, our results showed this function is conserved in mouse neurons. Notably, the syntaxin-1 LEAA mutant yielded limited rescue of Munc13-1–knockout mouse (Lai et al., 2017). We speculate that this difference is due to the expression of the C1C2BMUN NFAA mutant in cultured neurons instead of Munc13-1 knockout. The C1C2BMUN may have donated other sites than NF sequences in the interaction with Munc18-1/syntaxin-1. These other potential sites may interact with Q301/K308 of Munc18-1 domain 3a, which mediates the interaction between MUN and Munc18-1/syntaxin-1 (Wang et al., 2020). Besides, full-length Munc13-1 has additional functions beyond opening the syntaxin-1 linker region, such as membrane tethering and recruiting synaptobrevin-2 and SNAP-25 (Quade et al., 2019; Kalyana Sundaram et al., 2021).

Second, we investigated the relationship between the two conformational changes, and found that the domain 3a P335A mutant overcomes the requirement for syntaxin-1 RI sequences (Figures 4, 5), implying that the extension of domain 3a resulted from MUN interacting with the Munc18-1/syntaxin-1 complex, and is the subsequently step following the syntaxin-1 linker region opening. In addition, our previously experimental details need to be noted: (i) addition of the MUN domain enhanced the efficiency of SNARE complex assembly the transition when using Munc18-1 P335A but not when using syntaxin-1 LEAA; and (ii) single-molecule fluorescence resonance energy transfer (smFRET) experiments revealed that the linker region of syntaxin-1 adopted the closed conformation when bound to Munc18-1 P335A mutant. Altogether, we speculated that the opening of the syntaxin-1 linker region driven by the MUN domain is an early event, leading to the extension of domain 3a. The available data support the notion that the opening of the syntaxin-1 linker region leads to the extension of domain 3a (Wang et al., 2020). Similarly, the Munc18-1 P335A variant is equal to the function of syntaxin-1 RI sequences here, while in *C. elegans*, Munc18-1 P335A could partially rescued the phenotype of *unc-13* knockout, as previously reported (Park et al., 2017). Notably, in our previous study, Munc18-1 P335A partially overcame the SNARE assembly defect caused by loss of the MUN domain (Wang et al., 2020). This is likely because the syntaxin-1 RIAA mutant was expressed in our cultured neurons, which only abolished interaction with MUN NF sequences, not the absence of Munc13-1. This further suggests that other sites in the MUN domain are likely to be involved in the interaction with Munc18-1/syntaxin-1.

Third, we further explored the relationship between the syntaxin-1 linker region and Munc18-1 domain 3a, and found that once the syntaxin-1 linker is open, SNARE assembly and vesicle priming and fusion occur, no matter whether Munc18-1 domain 3a binds to the syntaxin-1 H3 (Figures 6, 7). According to previous results, the KKEE mutation in domain 3a abolishes SNARE complex assembly and vesicle release because of the inability of the variant to interact with the H3 domain of syntaxin-1 and template SNARE complex assembly. However, we found that the constitutively open form of the syntaxin-1 linker region fully rescued exocytosis in neurons, even when domain 3a was in an inactive state. We suspected that syntaxin-1 LEAA mutation in combination with the Munc18-1 KKEE mutation causes a strong effect on the conformation of syntaxin-1, leading to the escape of the H3 from Munc18-1 clamping and thus allowing the assembly in a way independent of Munc18-1 and Munc13-1.

The available data suggest that a conformational change in the syntaxin-1 linker region is key to initiate exocytosis and SNARE complex assembly. Unfortunately, we have not directly observed the dynamic conformation change in Munc18-1 domain 3a even with the single-molecule FRET experiments, because of its cysteine enrichment, which renders specific FRET-pair labeling difficult. It is hoped that with the future development of super-high-resolution microscopy, we will be able to observe the interactions and conformational changes of these key synaptic proteins directly and dynamically.

Overall, our results and those of previous studies illustrate the temporal and spatial relationships of the three synaptic proteins Munc13-1, Munc18-1, and syntaxin-1. First, Munc18-1 binds to syntaxin-1 to form the Munc18-1/syntaxin-1 complex, preventing SNARE complex assembly. The Munc13-1 MUN domain then interacts with the Munc18-1/syntaxin-1 complex to open the syntaxin-1 linker region. Subsequently, Munc18-1 domain 3a extends to template SNARE assembly.

## Data Availability Statement

The original contributions presented in the study are included in the article/Supplementary Material, further inquiries can be directed to the corresponding author/s.

## Ethics Statement

The animal study was reviewed and approved by the Animal Use Committee of South-Central University for Nationalities. Written informed consent was obtained from the owners for the participation of their animals in this study.

## Author Contributions

JG and CC performed the electro-physiological experiments. XW performed the biochemical experiments *in vitro*. XY and CM conceived the experiments. JG, XW, CC, YQ, and ZJ analyzed the data. JG wrote the manuscript. XW, XY, and CM revised the manuscript.

## Conflict of Interest

The authors declare that the research was conducted in the absence of any commercial or financial relationships that could be construed as a potential conflict of interest.

## Publisher’s Note

All claims expressed in this article are solely those of the authors and do not necessarily represent those of their affiliated organizations, or those of the publisher, the editors and the reviewers. Any product that may be evaluated in this article, or claim that may be made by its manufacturer, is not guaranteed or endorsed by the publisher.

## Supplementary Material

Supplementary Figure 1 The Munc18-1 P335A mutation supports the SNARE complex assembly from Munc18-1/syntaxin-1 with syntaxin-1 bearing RIAA mutation. The SNARE complex assembly from Munc18-1/syntaxin-1 RIAA (Munc18/Syx RI) or Munc18-1 P335A/syntaxin-1 RIAA mutant (Munc18 P335A/Syx RI) was detected by native gel in the presence of synaptobrevin-2 (Syb), SNAP-25 with or without MUN domain.
