# Supplementary figures and images for: Exploring the Two Coupled Conformational Changes That Activate the Munc18-1/Syntaxin-1 Complex
Source: Front Mol Neurosci. 2021 Dec 22;14:785696. doi: 10.3389/fnmol.2021.785696 (PMC8728020; doi:10.3389/fnmol.2021.785696)

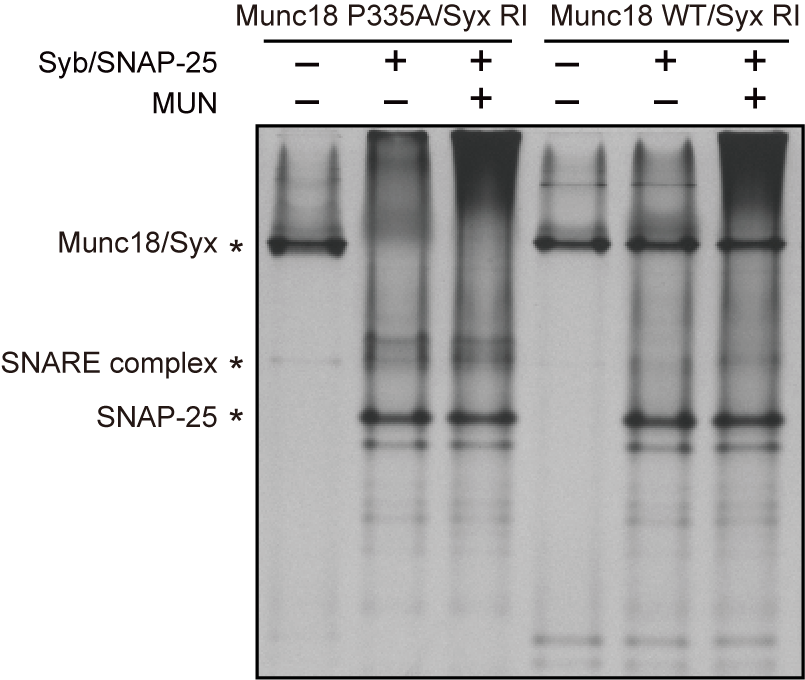

Supplement: Supplementary Figure 1 — The Munc18-1 P335A mutation supports the SNARE complex assembly from Munc18-1/syntaxin-1 with syntaxin-1 bearing RIAA mutation. The SNARE complex assembly from Munc18-1/syntaxin-1 RIAA (Munc18/Syx RI) or Munc18-1 P335A/syntaxin-1 RIAA mutant (Munc18 P335A/Syx RI) was detected by native gel in the presence of synaptobrevin-2 (Syb), SNAP-25 with or without MUN domain. [file Image_1.TIF]
